# Evaluation of Glove Liners Made of Semipermeable and Textile Materials in Patients With Hand Dermatoses: Results of a Multicenter Intervention Study (ProTection II)

**DOI:** 10.1111/cod.14822

**Published:** 2025-06-04

**Authors:** Theres Heichel, Jan F. Kersten, Antje Braumann, Kathrin Krambeck, Sonja Bonness, Claudia Schröder‐Kraft, Robert Ofenloch, Elke Weisshaar, Kerstin Strom, Christoph Skudlik, Flora Sonsmann, Annika Wilke, Albert Nienhaus, Swen Malte John, Richard Brans

**Affiliations:** ^1^ Institute for Interdisciplinary Dermatological Prevention and Rehabilitation (iDerm) at the Osnabrück University Osnabrück Germany; ^2^ Department of Dermatology, Environmental Medicine and Health Theory Osnabrück University Osnabrück Germany; ^3^ Competence Centre for Epidemiology and Health Service Research in Nursing, Institute for Health Service Research in Dermatology and Nursing (IVDP) University Medical Center Hamburg‐Eppendorf Hamburg Germany; ^4^ Institute for Interdisciplinary Dermatological Prevention and Rehabilitation (iDerm) at the Osnabrück University and BG Klinikum Hamburg Hamburg Germany; ^5^ Department of Dermatology Heidelberg University Hospital, Ruprecht Karls Universität Heidelberg Heidelberg Germany; ^6^ Department of Dermatology BG Hospital for Occupational Disease Bad Reichenhall Bad Reichenhall Germany; ^7^ Lower Saxony Institute of Occupational Dermatology University of Osnabrück Osnabrück Germany

**Keywords:** cotton, glove liner, hand eczema, occlusion, semipermeable, Sympatex, user acceptance, work‐related

## Abstract

**Background:**

Glove liners (GLs) are worn under impermeable gloves to prevent occlusion effects.

**Objectives:**

To evaluate the acceptance and tolerability of cotton gloves (COT) and semipermeable Sympatex gloves (SYM) as GLs in patients with hand dermatoses.

**Methods:**

In a prospective multicenter study, 210 patients with work‐related hand dermatoses were invited to wear either COT or SYM as GLs for 4 weeks underneath occlusive protective gloves during their regular work tasks. Acceptance of GL and health‐related quality of life (HRQoL) were assessed by questionnaires and the disease severity by the Osnabrueck Hand Eczema Severity Index.

**Results:**

A total of 178 data sets were available (SYM: *n* = 89/103, 86.4%; COT: *n* = 89/107, 83.2%). Both GLs did not impair work performance and were applicable in various work activities. SYM received better ratings regarding climate conditions, mobility and tactility. COT showed superiority in fit, donning and doffing. No substantial intergroup differences regarding disease severity and HRQoL were observed.

**Conclusions:**

SYM and COT were well tolerated and accepted, suggesting that SYM is a reasonable alternative for COT as GL in patients with hand dermatoses. The choice of material of GLs may depend on occupation‐specific requirements for the performed tasks, individual needs and preferences.

## Graphical abstract

SYM and COT were well tolerated in patients with hand dermatoses. SYM received better ratings regarding climate conditions, mobility and tactility, while COT showed superiority in fit, donning and doffing. The choice of material of glove liners may depend on occupation‐specific requirements for the performed tasks, individual needs and preferences. SYM is a reasonable alternative for COT as a glove liner in patients with hand dermatoses.

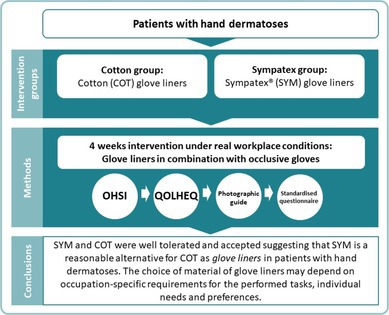

## 1 Introduction

In many occupations, gloves made of impermeable (watertight) materials (e.g., nitrile, latex) are worn to protect the skin from various hazards, including wetness, biohazards or chemicals and other irritants or allergens. This is primarily done to prevent infections and hand dermatoses, particularly, hand eczema, which is the most common occupational skin disease [1, 2, 3]. However, impermeable protective gloves induce occlusion effects, which in itself causes or worsens hand dermatoses, especially when these gloves are worn for prolonged periods of time in combination with the use of detergents or soaps, which is common in many occupations, including healthcare, cleaning, hairdressing or food handling [4]. To counteract the accumulation of moisture due to sweating and to prevent hand eczema, the additional use of textile glove liners (GLs) as separate glove‐like hand coverings is recommended when occlusive protective gloves are worn for more than 10 min [1, 2]. Usually, cotton (COT) gloves are used for this purpose. As the sweat absorbing capacity of textile GLs is limited, they must be replaced regularly by a dry pair when the material is saturated or soaked [1, 2].

Semipermeable gloves, such as Sympatex gloves (SYM), might be a good alternative to textile GLs. Sympatex is a semipermeable, non‐porous compact membrane consisting of a polyester–polyether copolymer which enables permeability of moisture (water vapour) along the diffusion gradient [5]. This process is controlled by the vapour pressure or temperature gradient between the inside and outside of the membrane [5]. If the membrane is used for GLs underneath impermeable protective gloves, dynamic climate regulation occurs between the skin surface, the membrane and/or the space between the gloves. The moisture produced by sweating is wicked away from the skin to the space between the gloves without reaching a saturation effect.

In an experimental study, we demonstrated previously that SYM and COT are able to counteract the delayed resolution of inflammation and epidermal barrier impairment caused by an occlusive glove material when applied as liners on pre‐irritated skin, suggesting a positive effect of SYM and COT when used as GLs in patients with hand dermatoses [6]. Recently, we conducted a quasi‐randomised trial in 120 healthcare workers with work‐related hand dermatoses to compare the tolerability and acceptance of SYM and COT when used as GLs underneath occlusive gloves for 10 ± 2 weeks under real workplace conditions [7]. The participants were recruited while taking part in a secondary individual prevention programme (SIP) for work‐related skin diseases (WRSDs). Both types of GLs were well tolerated and accepted by the participants. They were both suitable for various occupation‐specific activities and preferred wearing occlusive gloves without GLs. Given the previous study's limited scope, focusing on a specific occupational field, we have now executed a controlled multicenter study involving a larger number of patients with hand dermatoses from a broader spectrum of occupations, but keeping a focus on healthcare professionals. In contrast to the previous study, now patients taking part in a tertiary individual prevention programme (TIP) for WRSDs were recruited who usually have a more chronic and severe skin disease than patients taking part in the SIP and who have already more experience with prevention measures, including the use of GLs [8]. The primary objective of this study was to evaluate the acceptance and suitability of SYM and COT when used as GLs in different occupational settings by patients with hand dermatoses. Additionally, we aimed to assess the severity of the hand dermatoses and health‐related quality of life (HRQoL) over time.

## 2 Methods

### 2.1 Participants

As part of a research project (ProTection II), a controlled multicentre intervention study was conducted (sub‐study C), which was an extension of a previous study assessing the use of SYM/COT and COT as comfort gloves (sub‐studies A and B) [9]. As previously described, eligible adult patients with hand dermatoses insured by the Institution for Statutory Accident Insurance and Prevention in the Health and Welfare Services (Berufsgenossenschaft für Gesundheitsdienst und Wohlfahrtspflege, BGW) were enrolled at the start of a 3‐week inpatient phase of the TIP for severe WRSDs [8] after providing informed written consent. Recruitment was done between January 2020 and September 2022 in the four centres offering the TIP in Germany. Details of the recruitment criteria and process as well as the ethics approval have been published before (ethics committee of the University of Osnabrück, Germany, reference 4/71043.5; Medical Chamber of Lower Saxony, Hannover, Germany, reference Bo/08/2020) [9].

### 2.2 Intervention

As previously reported [9], eligible TIP patients willing to participate were assigned to one of the two intervention cohorts (COT or SYM) using a pre‐determined quasi‐randomised scheme established by the study coordination. They were invited to first participate in the intervention study assessing the use of SYM/COT and COT as comfort gloves overnight during the inpatient phase of the TIP (sub‐study A, T1–T4) and during the subsequent outpatient phase of the TIP while remaining absent from work (sub‐study B, T4–T5).

For sub‐study C, the participants were asked to wear COT or SYM as GLs underneath occlusive protective gloves after returning to work following the TIP during their regular work activities for 4 consecutive weeks. Only those who participated in sub‐study A were eligible for sub‐study C. Depending on the allocation of glove materials for sub‐studies A and B, each participant received the same gloves (either COT or SYM) for use as GLs in sub‐study C. Dropping out from one of the previous sub‐studies did not lead to exclusion from sub‐study C. The participants tested the GLs during the TIP to ensure a good fit when wearing them underneath the occlusive protective gloves, which were individually selected during the TIP and afterwards provided to the patients before returning to work. Each study participant received 50 pairs of COT‐GLs (multiple use, washable) or 220 pairs of SYM‐GLs (single use, not washable) including detailed instructions describing the specific use and care of the GLs (Table S1). As in the previous monocentric study [7], the participants were advised to use GLs when performing work tasks with occlusive protective gloves lasting > 10–15 min. For detailed information on the gloves (e.g., manufacturer, material strength), we refer to our previous publications [7, 9].

### 2.3 Assessments

Detailed information on the study procedure is presented in Table 1.

**TABLE 1:** Overview of the study procedure.

Phase as part of the tertiary individual prevention programme (TIP)	Sub‐study/study part	Time point (day)	Investigations/questionnaires
3‐week inpatient phase, including treatment, education and counselling as regular procedures of the TIP	(A) Wearing of comfort gloves overnight in inpatient setting (19 nights)	T1 (D2), T2 (D7), T3 (D14), T4 (D21), TX (D28)	OHSI 1 + OHSI 4 resp. OHSI X^a^ QOLHEQ (1) − QOLHEQ (2) PG (1) Questionnaire (1)
3‐week outpatient phase, including continued work absenteeism and treatment as regular procedure after the inpatient phase of the TIP	(B) Wearing of comfort gloves overnight in outpatient setting (at home) (19 nights)	T5 (D38)	OHSI 5 PG (2) Questionnaire (2)
4‐week period after return to work following the outpatient phase of the TIP	(C) Wearing of glove liners in occupational setting (4 weeks)	T6 (D71)	OHSI 6 QOLHEQ (3) PG (3) Questionnaire (3)

Abbreviations: OHSI, Osnabrueck Hand Eczema Severity Index; PG, photographic guide by Coenraads et al. [10]; QOLHEQ, Quality of Life in Hand Eczema Questionnaire; TIP, tertiary individual prevention programme.

^a^ Corresponds to an additional clinical investigation if there was a prolonged stay in TIP (general 1 week).

#### 2.3.1 Questionnaires to Assess Glove Use

Four weeks after return to work (T6), all study participants were provided with a slightly modified version of a paper‐based modularized questionnaire, which was developed in our department for previous studies and had been adjusted after pretests in patients with WRSDs [7, 11]. The questionnaire was sent to the participants by mail including a cover letter and a prepaid return envelope. In case of non‐response, a reminder was sent after 1 month. The questionnaire included the standardised recording of socio‐demographic characteristics (e.g., occupation) and an evaluation of the personal experience with the GLs, including ratings of statements on user acceptance and skin tolerability (e.g., ‘Under the gloves my skin feels moist.’) using a 4‐point Likert scale (Table 3). Other assessments (e.g., ‘wearing comfort’) were done by using a 6‐point scale based on the German school grading system (Table 4). We chose this scale because all participants are familiar with these categories. The participants were asked to rate the practicability for the activity they most frequently performed with the GLs during the study period. Further, the satisfaction with the GLs was assessed. Additional questions were used in order to determine if and how the COT‐GLs were cleaned and re‐used.

#### 2.3.2 Severity of Hand Dermatoses

As in sub‐studies A and B [9], the severity of hand dermatoses was evaluated by dermatologists using the Osnabrueck Hand Eczema Severity Index (OHSI) [12, 13].

For the follow‐up at T5 which was done during work absenteeism at the end of the outpatient phase before returning to work, all scores were included which were assessed between 14 and 28 days after T4 (end of inpatient phase) which corresponded to 89.6% of the data available for T5 (SYM: *n* = 87; COT: *n* = 77). For T6, all scores were included, which were assessed between 21 and 42 days after return to work (T5), which corresponded to 69.5% of the data available at T6 (SYM: *n* = 57; COT: *n* = 50).

The OHSI at T6 was compared with the OHSI at baseline (T1) and at T5. If participants were unable or unwilling to attend the follow‐up visit at the TIP centre, for instance, due to the long distance between their home and the centre, they were alternatively offered to attend a follow‐up at one of the BGW training and counselling centres (schu.ber.z) located throughout Germany, or at their treating dermatologist's office at home. Thus, the assessment at T6 was usually done by the same dermatologist who had already scored the OHSI at T5, but not necessarily the same one who had scored the OHSI at baseline (T1) [9].

Similarly to sub‐studies A and B [9], all participants were additionally asked to rate the severity of their hand dermatosis at T6 using the photographic guide [10, 14]. In patients with hand dermatoses other than hand eczema (e.g., palmar psoriasis), the OHSI and the photographic guide were applied in analogy to hand eczema.

#### 2.3.3 Health‐Related Quality of Life

The HRQoL was assessed after return to work (T6) using the Quality of Life in Hand Eczema Questionnaire (QOLHEQ) [15, 16, 17] as done during the previous phases of the research project in which the QOLHEQ was applied at baseline (T1) and at the end of the inpatient phase of the TIP (T4) [9].

### 2.4 Data Analyses

The data presentation comprises counts with corresponding rates for categorical data and mean values (*M*) with standard deviation (SD) for continuous variables. Fisher's exact test was employed to compare nominal scaled variables across different groups or time points. Additionally, the Cochran–Armitage test was applied for comparisons involving ordinal scales, for example, comparisons of the photographic guide. In the analysis of continuous scales, Student's *t* test was applied for comparisons involving two groups, while an analysis of variance (ANOVA) was utilised for comparisons of more than two groups and in case of baseline adjusted analyses. Drop‐out analyses pertaining to the initial intention to participate and the return of the questionnaire (T6) or taking part in the OHSI assessment (T6) were conducted using a binary logistic regression model with forward selection; the model incorporated potentially predictive variables that were initially present, including diagnosis (etiological component atopic/allergic/irritant, psoriasis), gender, group assignment, the OHSI score (T5) and the determined QOLHEQ (T4). Statistical analyses were conducted at a significance level of 5% using the SPSS software (IBM Corp. Released 2020. IBM SPSS Statistics for Windows, Version 27.0. Armonk, NY: IBM Corp) and the statistical software package R (R Core Team 2023, Vienna, Austria). No adjustment for multiplicity was conducted.

## 3 Results

### 3.1 Recruitment and Drop‐Outs

**FIGURE 1:**
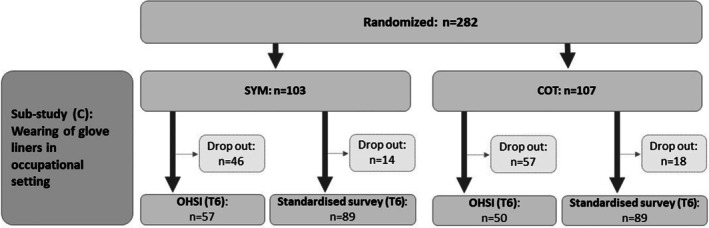
Flow chart of participants, Sympatex (SYM) and cotton (COT).

A total of 210 recruited patients expressed their willingness to participate in sub‐study C and were thus eligible to test the GLs made of SYM (*n* = 103, 70.5% of the patients originally enrolled in sub‐study A) or COT (*n* = 107, 78.7% of sub‐study A) underneath occlusive protective gloves over a period of 4 weeks (median [MD] 28 days) in everyday working life. At T6, questionnaires were returned by 178 participants (84.8%, drop out *n* = 32), among them 89 (86.4%) from the SYM cohort and 89 (83.2%) from the COT cohort (Table 2 and Figure 1). Fewer participants (*n* = 107, 51.0%, drop out *n* = 103) took part in the OHSI assessment at T6, among them 57 (55.3%) from the SYM cohort and 50 (46.7%) from the COT cohort (Table 2 and Figure 1).

**TABLE 2:** Participants' characteristics.

	Sympatex (SYM)	Cotton (COT)
Samples, *n*	103	107
Returned questionnaires, *n* (%)	89 (86.4)	89 (83.2)
Females, *n* (%)	83 (93.3)	82 (92.1)
Age in years, mean ± SD	47.9 ± 12.6	47.9 ± 12.0
Diagnosis		
Hand eczema, *n* (%)	85/89 (95.5)	83/89 (93.3)
Atopic hand eczema, *n* (%)	66 (74.2)	66 (74.2)
Irritant contact dermatitis, *n* (%)	62 (69.7)	75 (84.3)
Allergic contact dermatitis, *n* (%)	14 (15.7)	15 (16.9)
Other (*n*)	Palmar psoriasis (6) Palmar pustulosis (1) Dyshidrosis lamellosa sicca (1)	Palmar psoriasis (5) Palmar pustulosis (1) Lichen ruber (1) Pulpitis sicca (1)
Occupation, *n* (%)^a^		
Geriatric nurse	28 (31.5)	24 (27.6)
Hospital nurse	17 (19.1)	19 (21.8)
Hairdresser, beautician and foot care specialist	12 (13.5)	6 (6.9)
Housekeeper/cooks	8 (9.0)	7 (8.0)
Other health professionals	12 (13.5)	7 (8.0)
Physiotherapist, massage therapist etc.	5 (5.6)	10 (11.5)
Personal care workers in health services	3 (3.4)	6 (6.9)
Other occupations	4 (4.4)	8 (9.0)

Abbreviation: SD, standard deviation.

^a^ In each cohort, one participant worked in two occupations from two different occupational groups.

The majority of patients in both groups who returned the questionnaire were female, with an average age of about 50 years. The most common diagnosis in both groups was hand eczema (SYM: *n* = 85/89, 95.5%; COT: *n* = 83/89, 93.3%), most often consisting of combined etiologies (e.g., irritant contact dermatitis combined with atopic hand eczema). The most common occupations in both groups were hospital and geriatric nurses (Table 2).

In order to exclude systematic distortions in the sample by participants in sub‐study C who did not return the questionnaire at T6, a dropout analysis was performed. The analysis showed statistical significance solely in relation to age (OR = 1.5 95%‐CI: 1.09–2.07 per 10 years, *p* = 0.014). A similar dropout analysis regarding availability of OHSI values at T6 revealed again only a statistical significance for age (OR = 1.5; 95%‐CI: 1.07–2.06 per 10 years, *p* = 0.018). These results indicate that participants of advanced age exhibited an increased compliance in returning the questionnaire and in taking part in the OHSI assessment.

Most common reasons reported for interrupting or ceasing the application of GLs were: worsening of skin condition (SYM: *n* = 5/27), incapacity to work (SYM: *n* = 5/27), uncomfortable sweating sensation (SYM: *n* = 4/27; COT: *n* = 2/11), vacation (SYM: *n* = 3/27; COT: *n* = 2/11), changes in working hours (SYM: *n* = 2/27) and time pressure (SYM: *n* = 1/27; COT: *n* = 2/11).

In the SYM group, the average test duration was 24 days, with 60 individuals testing the GLs for at least 28 days (*M*: 24.5; MD: 28.0; range: 1–28 days; *n* = 77). In the COT group, the average test duration was also 24 days, with 76 individuals testing the GLs for at least 28 days (*M*: 27.6; MD: 28.0; range: 14–28 days; *n* = 79) (*p* < 0.001).

### 3.2 Application and Frequency of Use/Practicability Regarding Different Tasks

The average daily consumption for SYM‐GLs was 5.7 pairs (± 3.6; range: 1–20 pairs/day; *n* = 88) and for COT‐GLs 7.4 pairs (± 6.9; range: 1–50 pairs/day; *n* = 86) (*p* = 0.034). The work activities most frequently reported to be performed with GLs were similar in both groups (Table S2) with basic care of patients being the most frequent task, but the list also included, for example, hairdressing activities, housekeeping, cleaning or massage therapies. Both SYM‐GLs (*n* = 80/87, 92.0%) and COT‐GLs (*n* = 84/89, 94.4%) were used mainly in combination with disposable protective gloves. Most participants (SYM: 87.1%, *n* = 74/85; COT: 75.0%, *n* = 66/88) agreed that the (new) glove combination did not impair their ability to perform their work activities (*p* = 0.053). The majority of respondents agreed that the protective gloves were easy to put on over the GLs (SYM: 84.5%, *n* = 71/84; COT: 96.6%, *n* = 85/88; *p* = 0.008) and that the glove combinations could be taken off again without any problems (SYM: 89.2%, *n* = 74/83; COT: 92.1%, *n* = 82/89; *p* = 0.603). Perceived impairments in the use of the COT‐GLs mostly affected areas in which a high degree of tactility is required (*n* = 13/18). Impairments in the use of the SYM‐GLs were related to various issues (e.g., fit, *n* = 4/12; climate/perspiration/sweating, *n* = 2/12; additional time, *n* = 2/12).

### 3.3 Ratings of Properties of Gloves

SYM‐GLs scored significantly better than COT‐GLs in the categories ‘mobility of hands/fingers’ (*p* < 0.001) and ‘tactility’ (*p* < 0.001). ‘Climate’ including development of heat and moisture was rated better underneath SYM‐GLs than COT‐GLs (n.s.). SYM‐GLs scored better than COT‐GLs regarding ‘dry skin feeling even when wearing gloves for a long time’ (*p* = 0.002). COT‐GLs were rated significantly better than SYM‐GLs with regard to ‘fit’ (*p* < 0.001), ‘donning and doffing’ (*p* < 0.001) and ‘The material gives a comfortable wearing experience’. (*p* < 0.001). No statistically significant differences with regard to ‘suitability for everyday use’, ‘appearance’, ‘material feel on the skin’ and ‘wearing comfort’ between SYM and COT‐GLs were found (Tables 3 and 4). Most of the individual categories (e.g., wearing comfort), as well as the combinations of both GLs with occlusive gloves (SYM: M 2.53; COT: M 2.55, n.s.), were rated as ‘good to satisfactory’ (Table 4).

**TABLE 3:** Rating of cotton glove liners (COT) and Sympatex glove liners (SYM) after 4 weeks wear trial in occupational setting (T6).

Statement	Group/glove	*n*	*M*	SD	Differ‐ence	Lower limit	Upper limit	*p* (COT vs. SYM)
My hands stay dry even when wearing gloves for a long time.	COT	68	2.90	0.98	0.56	0.21	0.91	0.002.
	SYM	74	2.34	1.10				
Under the gloves my skin feels moist.	COT	77	2.77	0.89	−0.02	−0.35	0.31	0.906
	SYM	70	2.79	1.12				
My skin feels uncomfortably warm under the gloves.	COT	74	2.73	0.91	−0.25	−0.57	0.07	0.121
	SYM	59	2.98	0.96				
The gloves impair the mobility of my hand/fingers.	COT	70	2.70	1.00	−0.70	−1.05	−0.34	< 0.001
	SYM	43	3.40	0.82				
Movements can be executed purposefully with the gloves.	COT	82	2.41	0.90	0.53	0.24	0.82	< 0.001
	SYM	86	1.88	1.01				
The gloves strongly affect my sensitivity.	COT	81	2.20	0.98	−1.00	−1.31	−0.69	< 0.001
	SYM	61	3.20	0.89				
The gloves are easy to wear in everyday working life.	COT	87	2.05	0.89	0.06	−0.23	0.35	0.694
	SYM	83	1.99	1.03				
The material feels pleasant on the skin.	COT	89	1.52	0.69	−0.06	−0.28	0.15	0.565
	SYM	88	1.58	0.75				
The material gives a comfortable wearing experience.	COT	87	1.68	0.78	−0.48	−0.77	−0.20	< 0.001
	SYM	86	2.16	1.07				
The look of the gloves bothers me.	COT	37	2.78	1.32	−0.27	−0.84	0.30	0.351
	SYM	38	3.05	1.16				

*Note*: 4‐point Likert scale for agreement/satisfaction (1: strongly agree, 2: agree, 3: disagree, 4: strongly disagree).

Abbreviations: *M*, mean value; *n*, sample size; SD, standard deviation.

**TABLE 4:** Rating of cotton glove liners (COT) and Sympatex glove liners (SYM) after 4‐weeks wear trial in occupational setting (T6).

Category	Group/glove	*n*	*M*	SD	Differ‐ence	Lower limit	Upper limit	*p* (COT vs. SYM)
Fit/fitting	COT	88	1.82	0.62	−0.82	−1.09	−0.56	< 0.001
	SYM	89	2.64	1.10				
Wearing comfort	COT	88	1.91	0.87	−0.21	−0.50	0.07	0.136
	SYM	89	2.12	1.03				
Mobility/flexibility	COT	89	2.46	1.02	0.57	0.29	0.85	< 0.001
	SYM	89	1.89	0.86				
Tactility/tactile sensitivity	COT	86	3.20	1.34	1.13	0.77	1.49	< 0.001
	SYM	87	2.07	1.05				
Donning and doffing	COT	88	2.17	0.97	−0.85	−1.18	−0.52	< 0.001
	SYM	88	3.02	1.24				
Climate (perspiration/sweating)	COT	89	2.75	1.01	0.20	−0.14	0.54	0.240
	SYM	89	2.55	1.26				
Suitability for everyday use	COT	88	2.39	1.01	−0.20	−0.53	0.13	0.236
	SYM	89	2.58	1.19				
Overall/final evaluation combination	COT	89	2.55	0.92	0.02	−0.29	0.34	0.889
	SYM	89	2.53	1.21				

*Note*: Scale based on the German school grades system consisting of six numerical grades (1: very good, 2: good, 3: satisfactory, 4: sufficient, 5: poor, 6: very poor).

Abbreviations: *M*, mean value; *n*, sample size; SD, standard deviation.

### 3.4 Reprocessing of Cotton Glove Liners

A total of 10.2% (*n* = 9/88) of the participants from the healthcare sector reported having used the possibility of reprocessing the COT‐GLs in a professional textile laundry (workplace facility: *n* = 5, external service: *n* = 1, missing information: *n* = 3). Among them were three hospital nurses, two geriatric nurses, two cleaners, one housekeeper and one foot care specialist. One third of them stated that initiating this reprocessing process required substantial efforts (e.g., arrangements with supervisors) (37.5%, *n* = 3/8). The average frequency of professional cleaning with subsequent reuse of COT‐GLs was 8.1 times (range: 4–12.5 times, *n* = 4). The whole procedure of washing the GLs by a professional textile laundry took an average of 3.5 days (drop‐off to return) (range: 0.5–14 days, *n* = 6). The majority of participants reported having used their washing machine at home and regular washing detergents to clean the COT‐GLs (90.9%, *n* = 80/88), among them the majority of hospital nurses (*n* = 15/18) and geriatric nurses (*n* = 21/23). 60.8% (*n* = 48/79) put them in a special laundry bag and 26.9% washed them together with the private laundry (*n* = 21/78). The average frequency of cleaning with subsequent reuse with this method was 8.7 times (range: 1–100 times, *n* = 69). Six participants in the COT group used both types of washing procedures (professional textile laundry and washing machine at home) (6.8%, *n* = 6/88). More than three quarters of the participants stated that they had noticed changes in the material after washing (*n* = 79), including shrinking (49.4%, *n* = 39), change of touch or wearing comfort (40.5%, *n* = 32) and damage (e.g., holes, loose threads) (30.4%, *n* = 24).

### 3.5 Future Application and Glove Type Preferences

At the end of the questionnaire, the patients were asked about their future use of GLs. More than two thirds of the respondents in both cohorts stated that they were in favour of the routine use of the respective GLs at work (SYM: *n* = 57, 81.4%; COT: *n* = 74, 92.5%; n.s.), would recommend the respective GLs to others (SYM: *n* = 66, 90.4%; COT: *n* = 81, 100.0%; *p* = 0.005) and would also use them for domestic activities (SYM: *n* = 61, 76.2%; COT: *n* = 83, 97.6%; *p* < 0.001). Asked for glove type preference, the majority of respondents in the COT group stated that they would use COT‐GLs again (97.8%, *n* = 80/82), while 2.2% (*n* = 2) would not use GLs anymore. In the SYM group, 60.0% (*n* = 42/70) stated that they would use SYM‐GLs again (*n* = 42/70), while 32.9% (*n* = 23) would rather try other GLs (e.g., COT) and 7.1% (*n* = 5) would prefer not to use GLs in the future (Table S3).

### 3.6 Severity of Hand Dermatoses

#### 3.6.1 Osnabrück Hand Eczema Severity Index

**FIGURE 2:**
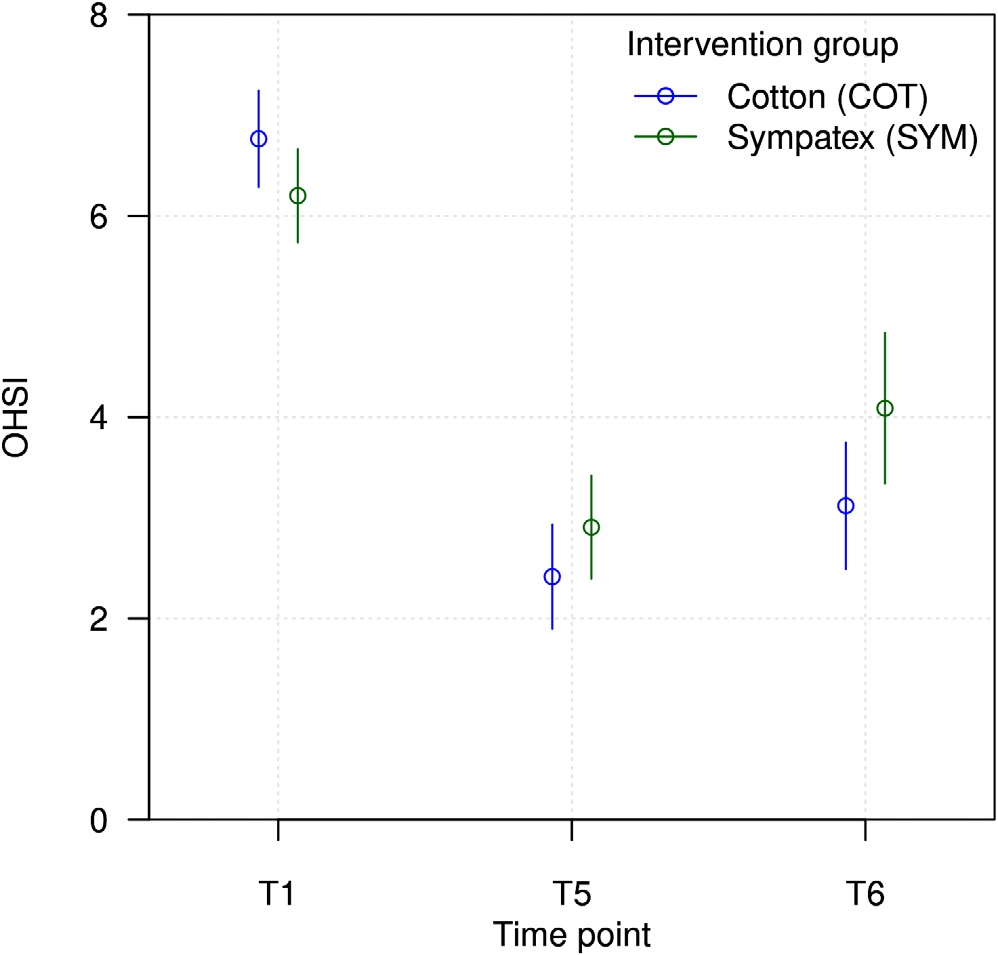
Mean values for the Osnabrueck Hand Eczema Severity Index (OHSI) of patients in the Sympatex (SYM) or cotton (COT) group at T1 (baseline, beginning of inpatient phase), T5 (end of 3‐week outpatient phase absent from work) and T6 (4 weeks after return to work).

On average, the skin condition of the hands worsened after return to work following the TIP, both when wearing SYM and COT as GLs. The OHSI increased from 2.99 (T5) to 4.09 (T6) points in the SYM cohort and from 2.44 (T5) to 3.14 (T6) points in the COT cohort (both *p* = 0.002) (Figure 2 and Table S4). There were no substantial differences between SYM and COT with respect to the development of the overall OHSI scores (n.s.). When analysing separately the six clinical signs assessed with the OHSI, worsening was observed in both groups (SYM and COT) after return to work with respect to erythema, scaling, infiltration and fissures (Figure S1 and Table S4). No substantial differences were observed between SYM and COT (n.s.). Notably, the OHSI values after return to work (T6) were still significantly lower than at baseline (T1) in both groups (SYM: 4.1 vs. 6.4, *p* < 0.001; COT: 3.1 vs. 6.3, *p* < 0.001). There were no statistically significant differences from T5 to T6 when comparing OHSI values of men and women (*p =* 0.875).

#### 3.6.2 Photographic Guide

At the end of the outpatient phase with absence from work (T5), the majority of patients of both groups rated their hand dermatosis at the more affected hand with ‘moderate’ to ‘(almost) clear’ (SYM: *n* = 60, 92.3%; COT: *n* = 69, 94.5%) (Table S5). Four weeks after return to work (T6), 78.5% (*n* = 51) of the SYM group and 86.3% (*n* = 63) of the COT group still described their skin condition as ‘moderate’ and better.

The self‐assessed severity worsened significantly in both intervention groups from T5 to T6 (Cochran‐Armitage tests for trend, SYM: *p* < 0.001; COT: *p* = 0.003). There were no statistically significant differences between the groups (T5: *p* = 1.0, T6: *p* = 0.3) in the classification ‘almost clear’ and better vs. ‘moderate’ and worse. A comparison of the initial values at baseline (T1) and the final values after return to work (T6) showed a significant improvement in both groups (SYM: *p* = 0.007; COT: *p* < 0.001; Fisher's exact test).

### 3.7 QOLHEQ

**FIGURE 3:**
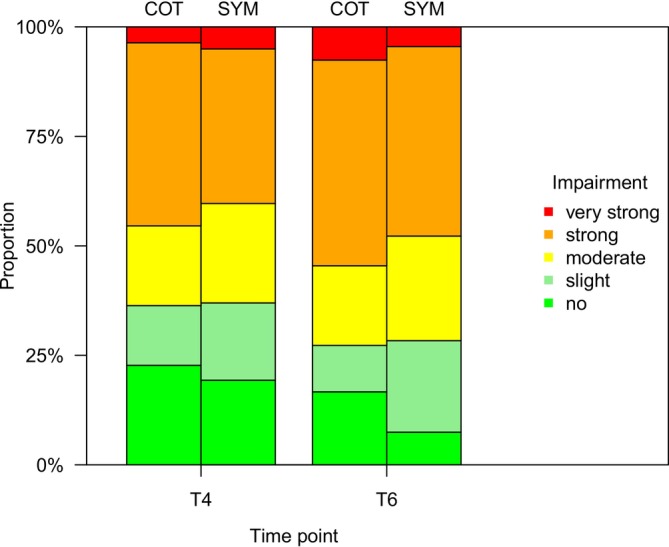
Health‐related quality of life impairment assessed with the Quality of Life in Hand Eczema Questionnaire (QOLHEQ) in the Sympatex (SYM) or cotton (COT) groups at T4 and T6: Interpretation of the overall QOLHEQ score.

In agreement with the clinical findings, there was a deterioration of the HRQoL as measured by the QOLHEQ 4 weeks after return to work following the TIP (T6) compared to the QOLHEQ at the end of the inpatient phase of the TIP (T4) (n.s.) (Figure 3 and Table S6).

The mean overall QOLHEQ score at T4 was 37.8 (± 21.8) in the SYM group and 38.9 (± 22.1) in the COT group, representing a moderately impaired HRQoL. At T6, an increase was observed, resulting in an overall score of 41.5 (± 23.1) in the SYM group and 45.4 (± 21.3) in the COT group, representing a moderate to strong impaired HRQoL (both n.s.). In the SYM group, the overall QOLHEQ score of patients with strong or very strong impairment changed from 40.3% (*n* = 48) at T4 to 47.8% (*n* = 32) at T6 (n.s.). In the COT group, the overall QOLHEQ score of patients also with strong or very strong impairment changed from 45.0% (*n* = 50) at T4 to 54.5% (*n* = 36) at T6 (n.s.).

Depending on the sub‐scales at T6, the highest impairment was found on the symptom subscale where 59.7% (*n* = 40, SYM) and 71.2% (*n* = 47, COT), respectively, of the patients were classified as having a ‘strong’ or ‘very strong’ HRQoL impairment in daily life because of symptoms. The emotion subscale represented a ‘slight’ to ‘moderate’ and the other QOLHEQ subdomains (functioning, treatment and prevention) a ‘moderate’ to ‘strong’ impaired HRQoL in both groups. There were no substantial differences between the groups with respect to the mean score of the QOLHEQ values and the mean values for subdomain scores (n.s.) (Figure S2, Tables S3 and S7). The only exception of this was the subdomain symptoms, which showed significantly higher values for SYM (12.1) compared to COT (9.5) at T6 (*p* = 0.026).

Notably, the total QOLHEQ scores after return to work (T6) were still significantly lower than at baseline (T1) in both groups (COT: 61.4 vs. 37.8; SYM: 60.3 vs. 45.4) (both *p* < 0.001).

## 4 Discussion

We here present the results of an intervention study in patients with hand dermatoses comparing the use of COT or SYM as GLs under workplace conditions after return to work following participation in a TIP for WRSDs.

### 4.1 Gloves Performance

The average test durations were comparable in both intervention groups, whereas the average daily consumption (gloves/day) was slightly but statistically significantly higher in the COT group. Nevertheless, the values indicate good comparability of the results between both groups and also underline the good applicability and tolerability of both SYM and COT.

In the present study, both types of GLs were frequently used in typical occupation‐specific activities and there was a high agreement that the GLs did not impair work performance under real working conditions. These results are in line with our previous study in healthcare workers [7] and other comparative studies [18, 19, 20] showing a good general suitability of both textile and semipermeable GLs for everyday use underneath protective gloves. For some aspects, ratings of SYM‐GLs and COT‐GLs differed substantially in the present study. SYM‐GLs were rated superior to COT‐GLs with regard to mobility (less impairment of mobility, better movement), tactility (less impairment) and dry skin feeling. COT‐GLs showed superiority to SYM‐GLs in fit, comfortable wearing experience, donning and doffing. There were only minor differences between the two types of GLs with regard to suitability for everyday use (easy to wear), appearance/look (impairment), climate (perspiration/sweating), material feel on the skin, wearing comfort, moist and warm feeling.

Overall, these results are in line with our previous study in healthcare workers who were recruited while taking part in the SIP [7]. Also in other studies, semipermeable GLs received better ratings for mobility and tactility than COT‐GLs [18, 21, 22]. In the present study, this is probably related to the about 5 times lower material thickness of SYM‐GLs (15 μm) compared to COT‐GLs (75 μm). This suggests that SYM‐GLs are superior to COT‐GLs for activities that require a high level of fine motor skills. However, regular use of COT‐GLs may lead to habituation effects reducing noticeable impairments of mobility and tactility [19, 23]. Otherwise, fingertip‐free or completely fingerless COT‐GLs could be an alternative in these cases.

In the present study, the climatic conditions under both GLs were rated as ‘good to satisfactory’ (Grades of 2–3). Also other studies showed that the climate conditions underneath occlusive gloves can be substantially improved when combined with textile [18, 19] and semipermeable [11, 18] GLs compared to wearing occlusive gloves alone. As the sweat absorbing capacity of COT‐GLs is limited, COT‐GLs must be regularly replaced by a dry pair when the material is saturated. In contrast, SYM‐GLs enable permeation of moisture along the diffusion gradient to the outer side of the membrane which is not subject to a saturation effect [5]. SYM‐GLs are therefore potentially more suitable when long continuous glove wearing is necessary. This may also explain why SYM‐GLs in comparison to COT‐GLs received better ratings for a dry skin feeling even when gloves are worn for a long time which was in line with our previous study [7].

As before [7], the fit and the wearing experience of COT‐GLs was rated better than that of SYM‐GLs which can be explained by the two‐dimensionality of the SYM‐GLs with a relatively loose fit. Similarly, SYM‐GLs received again worse ratings for their ‘donning and doffing’ behaviour. As discussed before [7], COT‐GLs prevent the adherence of the protective gloves to moist skin, which facilitates putting them on. The SYM‐GLs may also stick to moist skin and generate more friction under protective gloves, which may render it more difficult to put the SYM‐GLs on and to pull the occlusive gloves over them.

Some differences between both types of GLs noted in the previous study [7] were not detected anymore or were less pronounced. This might be related to differences in patients' characteristics of both studies or habituation effects. The participants of the previous study were asked to use the GLs right away in their work routine, whereas most of the participants in the present study got acquainted with them by using the gloves as comfort gloves in the sub‐studies A and B.

In the previous study in healthcare workers taking part in a SIP for prevention of WRSDs, the average daily consumption of SYM‐GLs was significantly higher than that of COT‐GLs, which we attributed to a potentially incorrect application (multiple use) of COT‐GLS [7]. The higher consumption of COT‐GLs than that of SYM‐GLs in the current study could be due to a more correct application of the GLs, as many of the TIP participants have longstanding hand dermatoses and are therefore already familiar with the use of COT‐GLs. The lower consumption of SYM‐GLs may indicate incorrect application (multiple use) in the present study, but could also indicate a lower level of satisfaction with this type of GLs.

### 4.2 Reprocessing

COT‐GLs could be reprocessed and re‐used several times, which was done by many participants of our study. A majority of them observed changes in the material after (multiple) washing, which mainly included shrinking, change of touch or wearing comfort and damage as seen by others [19]. Therefore, COT‐GLs must be replaced after a while. So far, there is a lack of generally accepted, specific recommendations for reprocessing COT‐GLs. However, particularly in healthcare or food handling, hygienic requirements must be followed. Therefore, cleaning of COT‐GLs in a professional textile laundry was recommended for these hygiene‐sensitive occupational settings. However, in the present study, the majority of participants, including healthcare workers, washed the COT‐GLs in their own household. This was in line with unpublished data from our previous study in healthcare workers [7], in which the majority of participants carried out the reprocessing of COT‐GLs at home (92.6%, *n* = 50/54) and only 5.7% (*n* = 3/55) used the possibility of reprocessing the COT‐GLs in a professional textile laundry. One possible reason for the small number of participants using a professional textile laundry despite working in hygiene‐sensitive occupations might be that in many workplaces, this process is not well‐established for COT‐GLs and might be accompanied with high implementation efforts, as reported by some participants. In contrast, cleaning COT‐GLs at home might be considered easier, likely underestimating related health risks. Moreover, participants who had already taken part in sub‐studies A and B likely used to clean COT at home after their application as comfort gloves and continued this procedure when wearing them as GLs in the present sub‐study C. Notably, uncertainties in how to change and reprocess GLs have been identified as a barrier to their (regular) use in healthcare workers [23, 24]. Taken together, this stresses the need for developing standardised recommendations for reprocessing COT‐GLs and establishing related procedures at workplaces which take hygienic requirements into account. This demand is addressed in another study (‘ProTection III’) [25].

### 4.3 Effects on Skin Condition and Health‐Related Quality of Life

The TIP is a well‐established intervention for patients with severe WRSDs in Germany. A multicenter study (ROQ) in 1788 patients evaluating the TIP demonstrated significant improvements of hand dermatoses from admittance until dismissal (OHSI: 6.3 to 2.8; *p* < 0.001) which lasted until the end of the subsequent outpatient phase, which can mostly be attributed to the interventions during the TIP, including intensified treatment and accompanying work absenteeism [26]. After return to work, the OHSI increased to 3.7 points in the ROQ study, corresponding to a slight deterioration most likely related to re‐exposure to occupational factors with a negative impact on the skin condition. The HRQoL, as measured by the Dermatology Life Quality Index (DLQI) showed a parallel course in that study. This was very similar in the present research project. In the first two sub‐studies A and B, in which the effect of wearing COT or SYM/COT as comfort gloves overnight was evaluated, an OHSI decrease was observed in both groups until the end of the inpatient phase of the TIP (T4, sub‐study A) and almost equalised until the end of the subsequent outpatient phase in which work absenteeism was continued (T5, sub‐study B) [9]. Here, we present the results of the follow‐up 4 weeks after return to work and having used SYM or COT as GLs during regular work activities (T6). The disease severity, as measured by the OHSI and the HRQoL, as measured by the QOLHEQ, deteriorated slightly after return to work in both groups. No substantial differences regarding the mean OHSI and QOLHEQ values were observed between the SYM and COT groups, suggesting that neither of the two materials was superior with regard to the disease course, including HRQoL and that both were similarly well tolerated. As in the ROQ study [26], the OHSI and QOLHEQ values at T6 were still significantly better compared to the corresponding values at the beginning of the TIP (T1). This suggests that the positive effect of the TIP on the skin condition persisted during the first weeks after return to work. The use of COT as GLs is already well‐established. Presumably, many of the TIP patients who took part in the previous ROQ study have used COT‐GLs, which may also partially explain the parallel development of the disease course and HRQoL outcome after return to work in both studies. A recent experimental study using bioengineering methods demonstrated that both SYM and COT are able to convey positive effects on pre‐irritated skin when applied as liners and counteract the delayed resolution of inflammation and epidermal barrier impairment caused by occlusive glove material [6]. This may have contributed to the positive outcome after return to work in the present study. However, the effects of the GLs cannot be assessed separately.

## 5 Limitations

In light of the randomised design employed in this study, it is essential to acknowledge the potential influence of non‐compliant patients on the outcomes. However, drop‐out analyses (regarding returning the questionnaire and participating in the OHSI assessment) showed only minor differences solely in terms of age. Even though we recruited participants with hand dermatoses from several occupations, most participants were healthcare workers. Therefore, it remains difficult to draw conclusions regarding employees working in other occupations and regarding individuals without hand dermatoses (and without resulting disease burden). In many TIP patients with chronic hand dermatoses, wearing COT‐GLs is already a well‐established prevention measure. Therefore, the evaluation of the GLs in the present study cannot be considered completely detached from these previous experiences. In addition, by taking part in the sub‐studies A and B, the participants got familiar with the gloves, which may have influenced their glove handling in the present study. Moreover, it is possible that participants who had a high level of satisfaction when using SYM or COT as comfort gloves in the sub‐studies A and B were more willing to continue with sub‐study C and were thus overrepresented. This may have led to an overestimation of the benefits of GLs.

It should be noted that the questionnaire on user acceptance, which was specifically designed to address the relevant questions, was not validated. However, as the questionnaire had been used already in a previous study and as similar results were obtained, this suggests a good reproducibility. Furthermore, the assessment tools used (OHSI, photographic guide, QOLHEQ) have not been validated for hand dermatoses other than hand eczema. However, as only a few participants suffered from other hand dermatoses (e.g., palmar psoriasis) and as these are in various aspects comparable to hand eczema, we consider this a minor limitation. Finally, some results are partly based on self‐reporting, which may have compromised accuracy (e.g., selective memory, attribution errors and exaggeration) [27].

## 6 Conclusion

In conclusion, our data indicates that both COT and SYM were suitable as GLs underneath occlusive gloves in patients with work‐related hand dermatoses when performing various occupation‐specific activities. SYM‐GLs were rated superior to COT‐GLs with regard to mobility, tactility and dry skin feeling even when gloves are worn for a long time. COT‐GLs showed superiority to SYM‐GLs in fit, comfortable wearing experience, as well as donning and doffing. For the other investigated aspects, including suitability for everyday use, appearance, material feel on the skin, wearing comfort, warm and moist skin feel under the gloves, no significant differences between both GLs were found. The study confirms that the GLs made of a semipermeable material serve as a reasonable alternative for COT‐GLs. The choice of material of GLs may depend on occupation‐specific requirements for the performed tasks and individual needs and preferences. The SYM‐GLs tested in the present study are prototypes and are not yet commercially available. For the time being, it is therefore difficult to compare the cost‐effectiveness of the two GLs. Our study indicates that there is an added value for GLs made of semipermeable materials in the prevention of work‐related hand dermatoses. Studies with longer follow‐up periods assessing the impact of varying severity of hand dermatoses would be of interest. In addition, future research should focus on hygienic (e.g., reprocessing), economic (e.g., costs) and environmental (e.g., sustainability) considerations regarding the application of GLs of different materials. Some of these aspects have already been addressed in another study (‘ProTection III’) [25].

## Author Contributions

**Theres Heichel:** conceptualization, data curation, formal analysis, funding acquisition, investigation, methodology, project administration, supervision, writing – review and editing, writing – original draft, visualization. **Jan F. Kersten:** writing – review and editing, writing – original draft, resources, software, methodology, formal analysis. **Antje Braumann:** investigation, project administration, writing – review and editing. **Kathrin Krambeck:** investigation, project administration, writing – review and editing. **Sonja Bonness:** investigation, project administration, writing – review and editing. **Claudia Schröder‐Kraft:** investigation, project administration, writing – review and editing. **Robert Ofenloch:** investigation, project administration, writing – review and editing. **Elke Weisshaar:** investigation, project administration, writing – review and editing. **Kerstin Strom:** investigation, project administration, writing – review and editing. **Christoph Skudlik:** conceptualization, funding acquisition, writing – review and editing. **Flora Sonsmann:** conceptualization, methodology, writing – review and editing. **Annika Wilke:** conceptualization, methodology, writing – review and editing. **Albert Nienhaus:** conceptualization, funding acquisition, writing – review and editing. **Swen Malte John:** conceptualization, funding acquisition, methodology, resources, supervision, writing – review and editing. **Richard Brans:** writing – original draft, writing – review and editing, conceptualization, funding acquisition, methodology, supervision.

## Conflicts of Interest

The authors declare no conflicts of interest.

## Supporting information


**Data S1.**Supporting Information.

## Data Availability

The data that support the findings of this study are available on request from the corresponding author. The data are not publicly available due to privacy or ethical restrictions.
