# Supplementary material for: Evaluation of Glove Liners Made of Semipermeable and Textile Materials in Patients With Hand Dermatoses: Results of a Multicenter Intervention Study (ProTection II)
Source: Contact Dermatitis. 2025 Jun 4;93(4):305–15. doi: 10.1111/cod.14822 (PMC12415674; doi:10.1111/cod.14822)
Supplement: Supplementary file 1 — Data S1.Supporting Information. [file COD-93-305-s001.pdf]

Appendices

Online Supplemental Figures

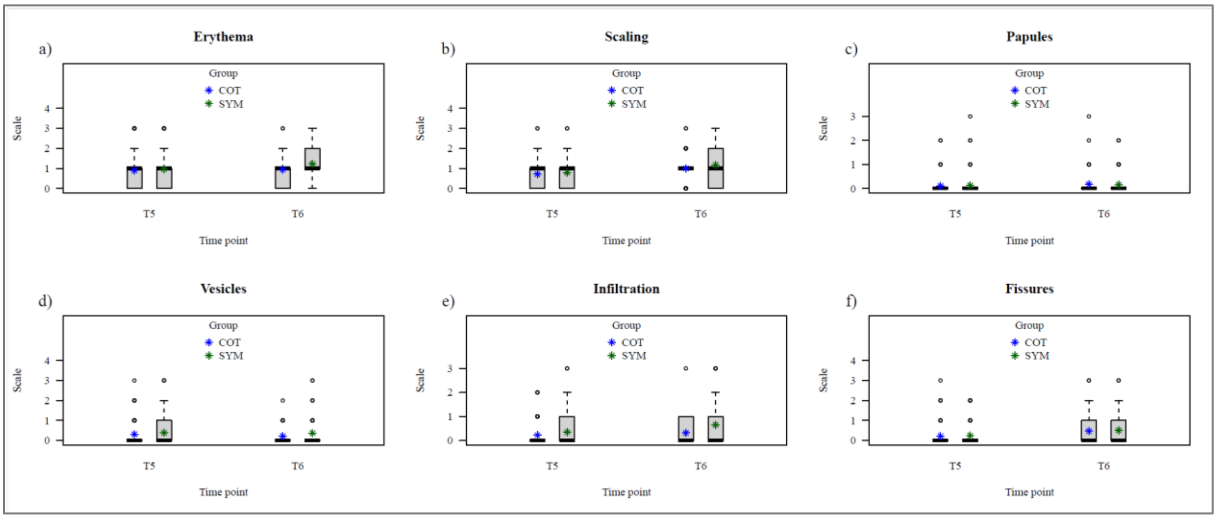

Online Supplemental Figure 1: Mean values for the OHSI (Osnabrueck Hand Eczema Severity Index) parameter a) erythema, b) scaling, c) papules, d) vesicles, e) infiltration and f) fissures of hands for intervention groups (cotton, COT; Sympatex, SYM) in the course of the study (T5 and T6).

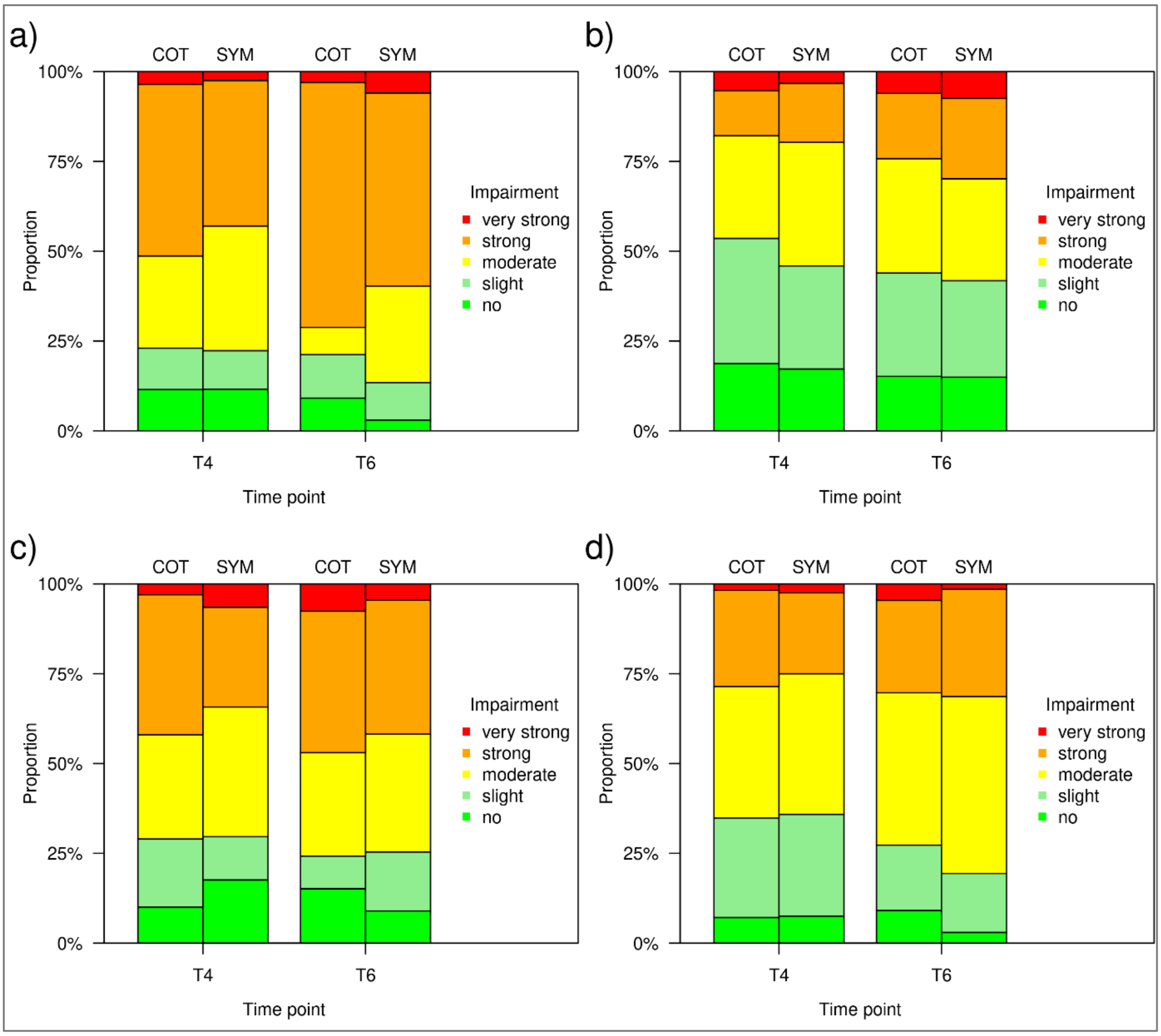

Online Supplemental Figure 2: Scores for the QOLHEQ (Quality of Life in Hand Eczema Questionnaire) subdomains a) symptoms, b) emotions, c) functioning, d) treatment and prevention for intervention groups (cotton, COT; Sympatex, SYM) in the course of the study (T4 and T6).

Online Supplemental Tables

Online Supplemental Table S1: Important informations and instructions for the gloves made of Sympatex (SYM) and cotton (COT).

Cotton glove (COT)	Sympatex glove (SYM)
▪ Use only for one task and replace immediately in case of heavy soiling, damage or contamination (e.g., due to tearing or puncturing of protective glove).<br>▪ Repeated use after re-processing possible.<br>▪ According to the manufacturer, the gloves can be washed at or below 60 °C or dry cleaning with perchloroethyelene (“P”) can be used. The gloves should not be bleached with chlorine, ironed or tumble dried.<br>▪ It is recommended to use a laundry bag.<br>▪ In hygiene-sensitive settings (e.g., healthcare), the gloves should be washed by a professional textile laundry.<br>▪ Disinfection by steam sterilization in an autoclave is recommended if sterile gloves are required.	▪ Single-use only.<br>▪ Discard after use.

Online Supplemental Table S2: Work activities for which the Sympatex (SYM) or cotton (COT) gloves liners were most frequently used.

Occupation	Activities	SYM [n]	COT [n]
Geriatric nurse<br>SYM: n = 28 (31.5 %)<br>COT: n = 24 (27.3 %)	Basic care (e.g., washing, bathing, showering, assistance with toilet visits, application of skin care products, incontinence care, pressure relief)	25	19
	Cleaning/instrument preparation/surface disinfection/sterilization	9	3
	Treatment care (e.g., administering medicine and drugs, applying surgical dressings, injections)	7	8
	Housekeeping / food exposure (e.g., food preparation, serving food, feeding)	5	2
	Unspecified/other (e.g., wet work, car driving, pushing wheelchair)	4	3
Hospital nurse<br>SYM: n = 17 (19.1 %)<br>COT: n = 19 (21.6 %)	Basic care (e.g., washing, bathing, showering, assistance with toilet visits, application of skin care products, incontinence care, pressure relief)	11	13
	Treatment care (e.g., administering medicine and drugs, applying surgical dressings, injections, collecting blood)	10	10
	Cleaning/instrument preparation/surface disinfection/sterilization	6	4
	Assistance (e.g., positioning patients, assistance in endoscopy)	1	1
	Housekeeping / food exposure	1	-
	Unspecified/other (e.g., administrative work, patient transportation)	-	2
Hairdresser, beautician and foot care specialist<br>SYM: n = 12 (13.5 %)<br>COT: n = 7 (8.0 %)	Hairdressing activities (e.g., cutting, washing, dyeing, bleaching and waving hair)	9	3
	Cosmetic / podological treatment	3	4
	Cleaning/instrument preparation/surface disinfection	2	2
Housekeeper/cooks<br>SYM: n = 8 (9.0 %)<br>COT: n = 7 (8.0 %)	Housekeeping (e.g., doing dishes, laundering, cleaning), food exposure (e.g., planning meals, preparing and cooking foodstuffs)	6	5
	Cleaning/instrument preparation/surface disinfection	2	6
Other health professionals (e.g., occupational therapist, medical assistant, dental assistant)<br>SYM: n = 12 (13.5 %)<br>COT: n = 7 (8.0 %)	Cleaning/instrument preparation/surface disinfection/sterilization	6	4
	Therapeutic activities (e.g., massage, assisting with mobility)	3	1
	Basic care	-	3
	Housekeeping	-	1
	Assistance during medical procedures (e.g., in endoscopy, x-rays, dental examinations, cleaning teeth)	5	2
	Treatment care (e.g., taking blood)	1	1
	Unspecified/other (e.g., lab work)	2	1
Physiotherapist, massage therapist etc.<br>SYM: n = 5 (5.6 %)<br>COT: n = 10 (11.4 %)	Physiotherapy, massage therapy and related treatments (e.g., massage, manual therapy, manual lymphatic drainage)	5	9
	Cleaning/instrument preparation/surface disinfection/sterilization	1	6
	Housekeeping / food exposure (e.g., washing up)	-	1
	Unspecified/other	-	1
	Housekeeping / food exposure (e.g., food preparation, serving food)	3	3

Personal care workers in health services<br>SYM: n = 3 (3.4 %)<br>COT: n = 6 (6.8 %)	Basic care (e.g., showering, assistance with toilet visits)	2	4
	Cleaning/instrument preparation/surface disinfection	1	3
	Unspecified/other (e.g., wet work)	-	2
**Other occupations**			
Pre-school teacher<br>SYM: n = 2 (2.2 %)<br>COT: n = 3 (3.4 %)	Cleaning/instrument preparation/surface disinfection	2	2
	Food exposure (e.g., food preparation)	1	1
	Unspecified/other (e.g., wet work, clean up/tidy up)	-	3
Cleaner<br>SYM: n = 1 (1.1 %)<br>COT: n = 4 (4.5 %)	Cleaning/instrument preparation/surface disinfection	1	4
Restaurant services workers<br>SYM: n = 1 (1.1 %)<br>COT: n = 1 (1.1 %)	Housekeeping (e.g., washing dishes, serving food)	1	1
	Cleaning/surface disinfection	1	-

Online Supplemental Table S3: Rating of cotton glove liners (COT) and Sympatex glove liners (SYM) regarding future application after 4-weeks wear trial in occupational setting (T6).

		Cotton group (COT)	Sympatex group (SYM)
Routine use of the respective glove liner at work	Yes [n, rate]	74 (86.0%)	57 (65.5%)
	No [n, rate]	6 (7.0%)	13 (14.9%)
	Don't know [n, rate]	6 (7.0%)	17 (19.5%)
Recommend the respective glove liner to others	Yes [n, rate]	81 (96.4%)	66 (75.9%)
	No [n, rate]	0 (0.0%)	7 (8.0%)
	Don't know [n, rate]	3 (3.6%)	14 (16.1%)
Use glove liner for domestic activities	Yes [n, rate]	83 (97.6%)	61 (70.1%)
	No [n, rate]	1 (1.2%)	19 (21.8%)
	Don't know [n, rate]	1 (1.2%)	7 (8.0%)
Favorite glove /combination for use as glove liners	No glove liner (only protectice gloves)	2 (2.2%)	5 (7.1 %)
	Combination with sympatex	(not applicable)	42 (60.0 %)
	Combination with cotton	80 (97.8%)	23 (32.9 %)

Abbreviations: n, sample size

Online Supplemental Table S4: Results of the Osnabrueck Hand Eczema Severity Index (OHSI) and its individual clinical signs for the Sympatex (SYM) and cotton group (COT) in the course of the study (T5 and T6).

Parameters	Glove type / group	T5			T6		
		M	SD	n	M	SD	n
**OHSI**	SYM	2.99	2.52	87	4.09	2.88	57
	COT	2.44	2.24	77	3.14	2.27	50
**Erythema**	SYM	0.97	0.94	86	1.23	0.91	57
	COT	0.90	0.99	77	0.94	0.76	51
**Scaling**	SYM	0.79	0.81	86	1.18	0.95	57
	COT	0.71	0.76	77	1.00	0.75	51
**Papules**	SYM	0.13	0.48	86	0.16	0.45	57
	COT	0.10	0.38	77	0.18	0.56	51
**Vesicles**	SYM	0.38	0.72	86	0.37	0.75	57
	COT	0.31	0.65	77	0.22	0.46	51
**Infiltration**	SYM	0.35	0.67	86	0.65	0.90	57
	COT	0.22	0.53	77	0.31	0.58	51
**Fissures**	SYM	0.24	0.59	86	0.51	0.85	57
	COT	0.22	0.60	77	0.47	0.83	51

Abbreviations: M, mean value; ±SD, standard deviation; n, sample size

Online Supplemental Table S5: Photographic guide for the Sympatex (SYM) and cotton group (COT) in the course of the study (T5 and T6) for participants for whom both statements were available.

		Cotton group (COT)		Sympatex group (SYM)	
		T5	T6	T5	T6
Hand eczema at present, more affected hand	Very severe [n, %]	1 (1.4 %)	3 (4.1 %)	1 (1.5%)	1 (1.5%)
	Severe [n, %]	3 (4.1 %)	7 (9.6 %)	4 (6.2%)	13 (20.0%)
	Moderate [n, %]	18 (24.7 %)	25 (34.2 %)	9 (13.8%)	23 (35.4%)
	Almost clear [n, %]	29 (39.7 %)	30 (41.1 %)	36 (55.4%)	22 (33.8%)
	Clear [n, %]	22 (30.1 %)	8 (11.0 %)	15 (23.1%)	6 (9.2%)

Abbreviations: n, sample size

Online Supplemental Table S6: Health-related quality of life impairment assessed with the Quality of Life in Hand Eczema Questionnaire (QOLHEQ) in patients with hand eczema: Presented are the mean values (M) with standard deviations (SD) of the overall score and its four subdomains for the Sympatex (SYM) and cotton group (COT) in the course of the study (T4 and T6).

QOLHEQ	Cotton group (COT)		Sympatex group (SYM)	
	T4 (M, SD)	T6 (M, SD)	T4 (M, SD)	T6 (M, SD)
**Overall score**	38.9 (22.1)	37.8 (21.8)	41.5 (23.1)	45.4 (21.3)
**Symptoms**	10.1 (5.7)	9.5 (5.5)	11.0 (5.5)	12.1 (5.4)
**Emotion**	8.9 (6.5)	9.3 (6.5)	9.6 (6.8)	11.1 (6.6)
**Functioning**	10.3 (6.9)	9.6 (7.0)	10.4 (7.2)	10.8 (6.8)
**Treatment and prevention**	10.0 (5.2)	9.8 (5.3)	10.5 (5.4)	11.5 (4.6)

Abbreviations: M, mean value; ±SD, standard deviation

Note: Overall score: 0-17 no impairment, 18-28 slight impairment, 29-41 moderate impairment, 42-79 strong impairment, 79-117 very strong impairment.
Subdomain 'symptoms': 0-2 no impairment, 3-5 slight impairment, 6-9 moderate impairment, 10-20 strong impairment, 21-27 very strong impairment.
Subdomain 'emotions': 0-2 no impairment, 3-8 slight impairment, 9-14 moderate impairment, 15-21 strong impairment, 22-31 very strong impairment.
Subdomain 'functioning': 0-2 no impairment, 3-5 slight impairment, 6-11 moderate impairment, 12-23 strong impairment, 24-32 very strong impairment.
Subdomain 'treatment and prevention': 0-2 no impairment, 3-7 slight impairment, 8-13 moderate impairment, 14-20 strong impairment, 21-27 very strong impairment.

Online Supplemental Table S7: Health-related quality of life impairment assessed with the Quality of Life in Hand Eczema Questionnaire (QOLHEQ) in patients with hand eczema: Presented are the proportions of participants for each grading in the Sympatex (SYM) and cotton group (COT) of the overall score and the four subdomains at in the course of the study (T4 and T6).

QOLHEQ		Cotton group (COT)		Sympatex group (SYM)	
		T4	T6	T4	T6
**Overall score**	Not at all [n, %]	25 (22.7%)	11 (16.7%)	23 (19.3%)	5 (7.5%)
	Slightly [n, %]	15 (13.6%)	7 (10.6%)	21 (17.6%)	14 (20.9%)
	Moderately [n, %]	20 (18.2%)	12 (18.2%)	27 (22.7%)	16 (23.9%)
	Strongly [n, %]	46 (41.8%)	31 (47.0%)	42 (35.3%)	29 (43.3%)
	Very strongly [n, %]	4 (3.6%)	5 (7.6%)	6 (5.0%)	3 (4.5%)
**Symptoms**	Not at all [n, %]	13 (11.5%)	6 (9.1%)	14 (11.6%)	2 (3.0%)
	Slightly [n, %]	13 (11.5%)	8 (12.1%)	13 (10.7%)	7 (10.4%)
	Moderately [n, %]	29 (25.7%)	5 (7.6%)	42 (34.7%)	18 (26.9%)
	Strongly [n, %]	54 (47.8%)	45 (68.2%)	49 (40.5%)	36 (53.7%)
	Very strongly [n, %]	4 (3.5%)	2 (3.0%)	3 (2.5%)	4 (6.0%)
**Emotions**	Not at all [n, %]	21 (18.8%)	10 (15.2%)	21 (17.2%)	10 (14.9%)
	Slightly [n, %]	39 (34.8%)	19 (28.8%)	35 (28.7%)	18 (26.9%)
	Moderately [n, %]	32 (28.6%)	21 (31.8%)	42 (34.4%)	19 (28.4%)
	Strongly [n, %]	14 (12.5%)	12 (18.2%)	20 (16.4%)	15 (22.4%)
	Very strongly [n, %]	6 (5.4%)	4 (6.1%)	4 (3.3%)	5 (7.5%)

**Functioning**	Not at all [n, %]	10 (10.0%)	10 (15.2%)	19 (17.6%)	6 (9.0%)
	Slightly [n, %]	19 (19.0%)	6 (9.1%)	13 (12.0%)	11 (16.4%)
	Moderately [n, %]	29 (29.0%)	19 (28.8%)	39 (36.1%)	22 (32.8%)
	Strongly [n, %]	39 (39.0%)	26 (39.4%)	30 (27.8%)	25 (37.3%)
	Very strongly [n, %]	3 (3.0%)	5 (7.6%)	7 (6.5%)	3 (4.5%)
**Treatment and prevention**	Not at all [n, %]	8 (7.1%)	6 (9.1%)	9 (7.5%)	2 (3.0%)
	Slightly [n, %]	31 (27.7%)	12 (18.2%)	34 (28.3%)	11 (16.4%)
	Moderately [n, %]	41 (36.6%)	28 (42.4%)	47 (39.2%)	33 (49.3%)
	Strongly [n, %]	30 (26.8%)	17 (25.8%)	27 (22.5%)	20 (29.9%)
	Very strongly [n, %]	2 (1.8%)	3 (4.5%)	3 (2.5%)	1 (1.5%)

Abbreviations: n, sample size
